# The long non-coding RNA NEAT1 enhances epithelial-to-mesenchymal transition and chemoresistance via the miR-34a/c-Met axis in renal cell carcinoma

**DOI:** 10.18632/oncotarget.17757

**Published:** 2017-05-10

**Authors:** Fei Liu, Na Chen, Yanchun Gong, Ruihai Xiao, Weichao Wang, Zhengyue Pan

**Affiliations:** ^1^ Department of Urology, The Second Affiliated Hospital of Nanchang University, Nanchang 330006, China; ^2^ Department of Breast Surgery, The Fourth Affiliated Hospital of Nanchang University, Nanchang 330003, China; ^3^ School of Life Science, Jiangxi Science and Technology Normal University, Nanchang 330013, China

**Keywords:** renal cell carcinoma, NEAT1, miR-34a, c-Met, chemotherapy

## Abstract

Long non-coding RNAs (lncRNAs) have emerged as new gene regulators and prognostic markers in various cancers. Although the lncRNA nuclear enriched abundant transcript 1 (NEAT1) has been associated with tumorigenesis, its functions in renal cell carcinoma (RCC) have not been elucidated. We determined that NEAT1 is up-regulated in RCC tissue compared to corresponding non-tumor tissue. High NEAT1 expression was associated with tumor progression and poor survival in RCC patients. NEAT1 knockdown suppressed RCC cell proliferation by inhibiting cell cycle progression, and inhibited RCC cell migration and invasion by reversing the epithelial-to-mesenchymal transition phenotype. Down-regulation of NEAT1 increased the sensitivity of RCC cells to sorafenib *in vitro*. Mechanistic analysis revealed that NEAT1 acts as a competitive sponge for miR-34a, which prevents inhibition of c-Met. Thus, NEAT1 promotes RCC progression through the miR-34a/c-Met axis.

## INTRODUCTION

Renal cell carcinoma (RCC) accounts for nearly 90% of all kidney cancers [1, 2]. Approximately 20% of RCC patients are diagnosed at an advanced stage, and 30% of patients with localized RCC develop metastasis or local recurrence following nephrectomy [3]. Patients with advanced RCC typically respond poorly to conventional treatment with chemotherapy and radiotherapy [4]. A better understanding of the molecular mechanisms underlying RCC progression, including metastasis and drug resistance, is required to improve RCC treatment.

Long non-coding RNAs (lncRNAs) are a new class of non-protein coding RNAs with a minimum length of 200 nucleotides [5]. LncRNAs have been implicated in multiple biological processes including cell differentiation, apoptosis, epigenetic regulation of gene expression, and RNA decay [6, 7]. Recently, several lncRNAs have been shown to have crucial roles in carcinogenesis, metastasis, and drug resistance [8, 9].

The nuclear paraspeckle assembly transcript 1 (NEAT1) is a newly identified lncRNA that regulates gene expression by retaining mRNAs for editing in the nucleus [10]. Aberrant NEAT1 expression has been has been observed in several human cancers [11, 12]. For example, over-expression of NEAT was associated with tumor recurrence and an unfavorable prognosis in colorectal cancer [13]. Recently, Li et al. demonstrated that NEAT1 induced epithelial-to-mesenchymal transition (EMT) and promoted 5-fluorouracil resistance in breast cancer [14]. However, the role of NEAT1 in RCC is still unclear.

Here, we show that NEAT1 expression is up-regulated in RCC tissues and is correlated with poor patient prognosis. Down-regulation of NEAT1 inhibits growth, cell cycle progression, and metastasis in RCC, and enhances the sensitivity of malignant cells to sorafenib. Our data demonstrate that NEAT1 acts as a miRNA sponge to directly suppress miR-34a, and promotes RCC progression through inhibition of the miR-34a/c-Met axis. Thus, NEAT1 may be serve as a prognostic biomarker and therapeutic target in RCC.

## RESULTS

### NEAT1 is up-regulated in RCC tissue and cell lines, and is correlated with poor prognosis

**Figure 1:**
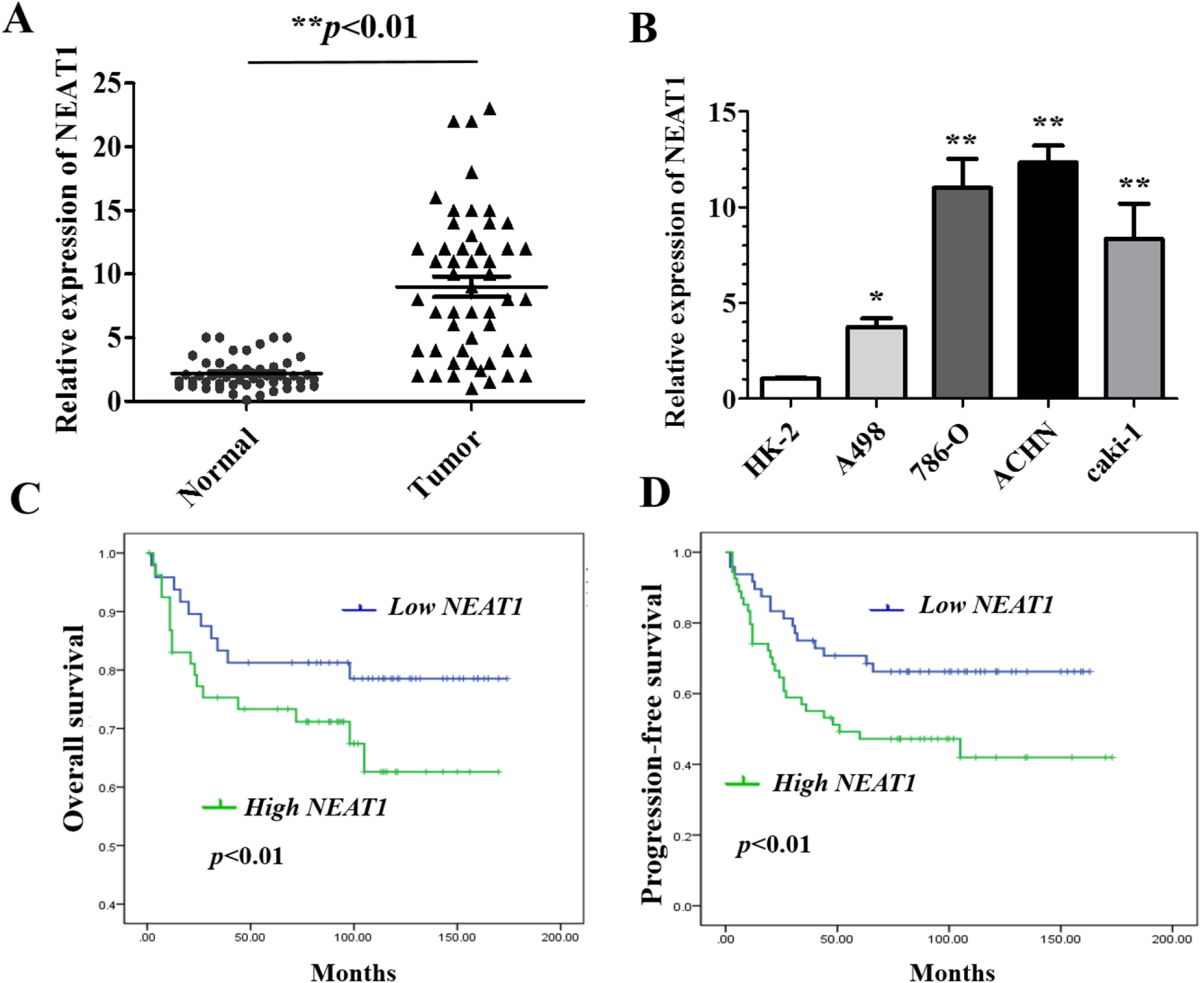
Relative NEAT1 expression in RCC tissue and cell lines, and its clinical significance **(A)** Relative expression of NEAT1 expression in RCC (n = 102) and paired normal (n =102) tissue samples. NEAT1 expression was analyzed by qRT-PCR and normalized to GAPDH expression. Statistically significant differences between samples were identified using paired t-tests (n = 102, * *p* < 0.01). **(B)** Analysis of NEAT1 expression in four RCC cell lines and HK-2 cells (mean ± SEM).*p < 0.05 and *p < 0.01, respectively. **(C** and **D)** Kaplan-Meier curves for overall survival **(C)** and progression-free survival **(D)** in RCC patients with low and high NEAT1 expression.

We first evaluated NEAT1 expression in paired RCC and adjacent normal human tissue samples from RCC patients. As expected, NEAT1 expression was significantly higher in RCC compared to adjacent normal tissue (Figure 1A, *p* < 0.01). We next assessed NEAT1 expression in four different RCC cell lines with varying malignant potential (ACHN, 786-O, A498, Caki-1) relative to normal epithelial kidney cells (HK-2). NEAT1 expression was higher in the four RCC cell lines than in normal HK-2 cells (*p* < 0.05 and *p* < 0.01, respectively) (Figure 1B). We selected two representative RCC cell lines with high NEAT1 expression, one primary (786-O) and one metastatic (ACHN), for functional studies.

To explore the clinical significance of NEAT1 in RCC, we analyzed the association between NEAT1 expression and the clinicopathological characteristics of 102 RCC patients. These results indicated that NEAT1 expression in RCC tissue was significantly correlated with TNM stage, tumor size, and the presence of lymph node metastasis, but not with age, sex, or tumor location (Table 1). We also investigated the relationship between NEAT1 expression and RCC patient prognosis. Kaplan-Meier analysis revealed that patients with high NEAT1 expression had poorer overall (*p* < 0.01) and progression-free survival (*p* < 0.01) than those with low NEAT1 expression (Figure 1C and 1D). Collectively, these data suggest that up-regulation of NEAT1 may contribute to RCC progression and that it is a prognostic biomarker in RCC.

**Table 1:** Relationship between NEAT1 expression and clinicopathological features

Parameters	n	NEAT1 expression		*P* value
		Low(n=34)	High(n=68)	
Age (years)				*P*=0.701
>56	54	17	37	
≤56	48	17	31	
Gender				*P*=0.371
Female	42	15	37	
Male	60	19	31	
Location				*P*=0.392
Left	55	16	39	
Right	47	18	29	
TNM stage				P<0.01**
T1-T2	33	23	11	
T3-T4	68	11	57	
Lymph node metastasis				P<0.01**
Yes	42	6	36	
No	60	28	32	
Tumor size(cm)				P<0.01**
>7	48	8	40	
≤7	54	26	28	
Recurrence				*P*=0.169
Yes	49	20	29	
NO	53	14	39	

### NEAT1 knockdown inhibits cell growth and cell cycle progression of RCC cells *in vitro*

**Figure 2:**
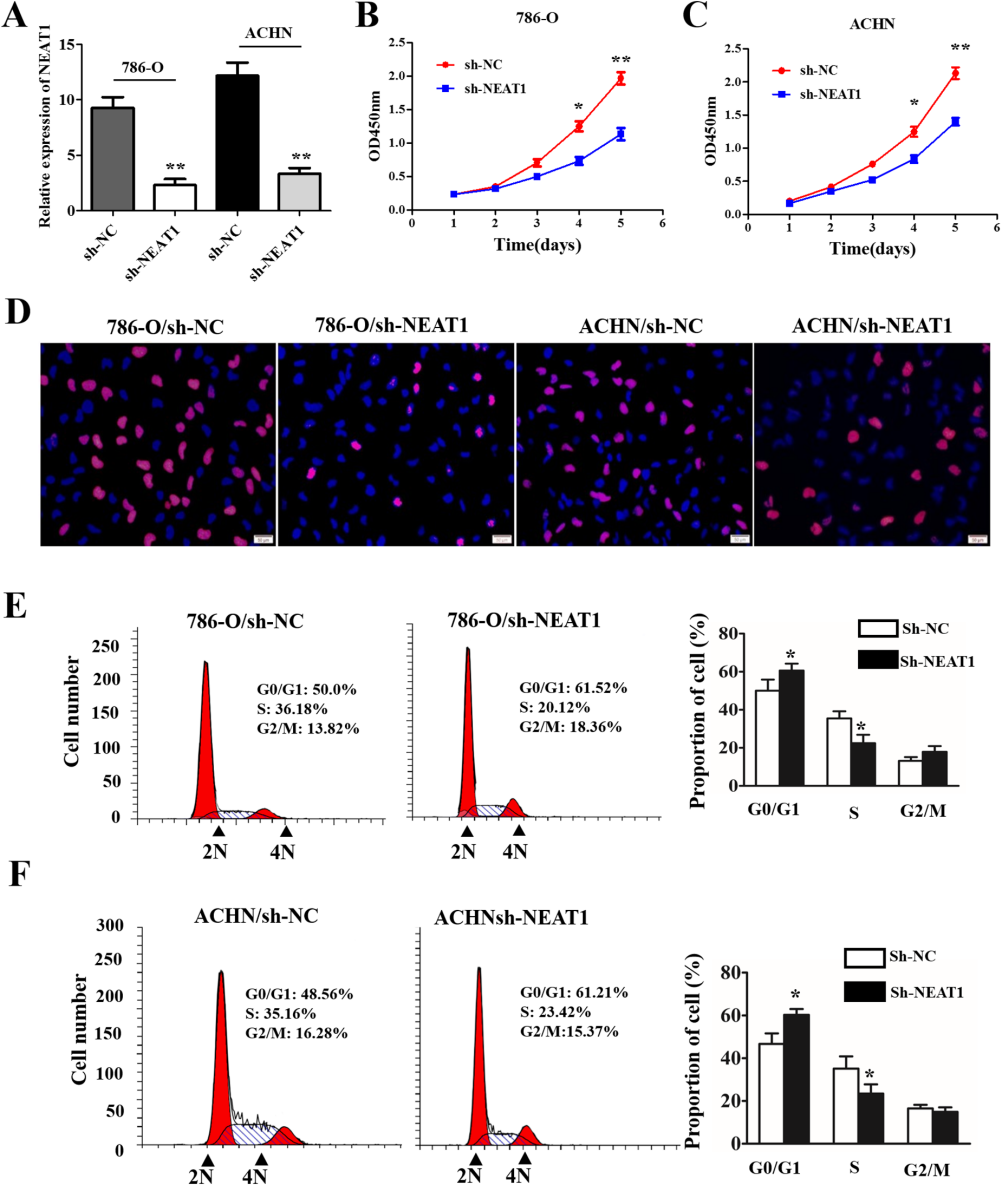
The effects of NEAT1 on RCC cell proliferation and cell cycle progression **(A)** The efficiency of NEAT1 silencing in RCC cell lines measured by qRT-PCR. **(B** and **C)** Representative results of CCK-8 cell proliferation assays. **(D)** Representative photomicrographs of EdU staining in 786-O and AHCN cells after transfection with shNEAT1. The click-iT reaction revealed EdU staining (red). Cell nuclei are stained with DAPI. **(E** and **F)** Analysis of cell cycle progression in RCC cells (786-O and AHCN) following NEAT1 knockdown. Peak diagram (left) and distribution of cells in the G0/G1, S, and G2/M phases of the cell cycle (right).*p < 0.05 and *p < 0.01, respectively, compared to controls.

To further explore the oncogenic roles of NEAT1 in RCC, we established stable NEAT1 knockdown cell lines (786-O and ACHN) by transfecting RCC cells with NEAT1 shRNA (Figure 2A; *p* < 0.01). CCK-8 assays demonstrated that NEAT1 knockdown inhibited the proliferation of 786-O and A498 cells (Figure 2B and 2C). In addition, EdU staining indicated that NEAT1 knockdown suppressed DNA synthesis in RCC cells compared to controls (Figure 2D). We next investigated the effects of NEAT1 knockdown on cell cycle progression. NEAT1 knockdown by shRNA resulted in the accumulation of cells in the G0/G1 phase, and a decrease in the number of cells in S-phase compared to controls (Figure 2E and 2F, p < 0.05).

### NEAT1 knockdown suppresses cell migration and invasion by reversing the EMT phenotype

**Figure 3:**
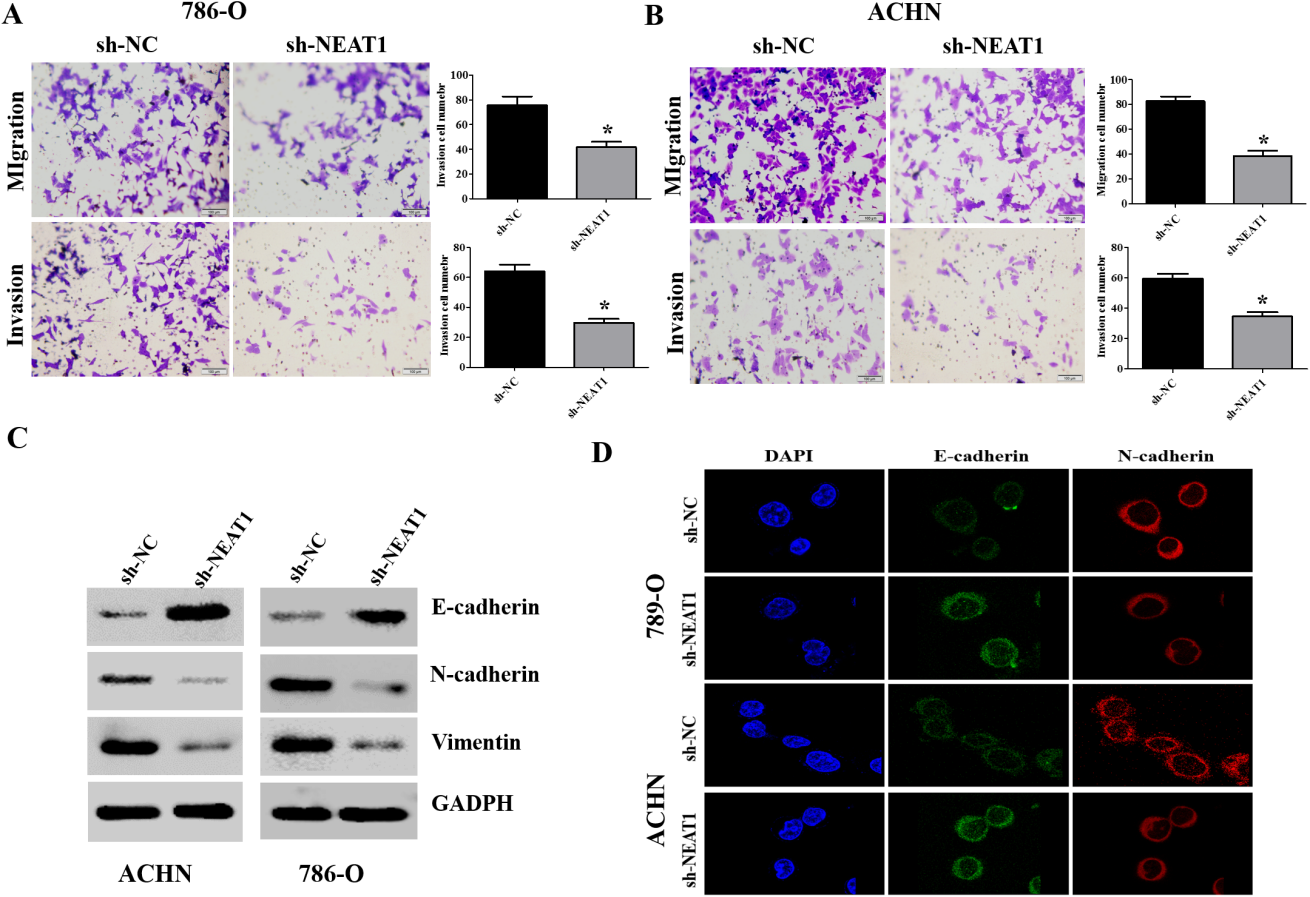
NEAT1 promotes EMT, migration, and invasion in RCC cells **(A** and **B)** Representative results of the migration and Transwell invasion assays showing the effect of NEAT1 knockdown on the migratory abilities of 786-O and AHCN cells (unpaired Student's t-test, mean ± SEM; *p < 0.05). **(C)** Western blot analysis of phenotypic markers including E-cadherin, N-cadherin, and vimentin in NEAT1 knockdown cells. GAPDH was used as the loading control. **(D)** Confocal microscopy analysis of the expression of E- and N-cadherin. The red signal represents E- or N-cadherin staining and the blue signal represents nuclear DNA staining (DAPI).

Next, we investigated the effects of NEAT1 on RCC cell migration and invasion. NEAT1 knockdown suppressed 786-O and ACHN cell migration and invasion (Figure 3A and 3B, p < 0.05). Because EMT is a critical event in metastasis [17], we investigated whether NEAT1 knockdown could reverse the EMT phenotype in RCC cells. Western blot analysis indicated that knockdown of NEAT1 in 786-O and ACHN cells resulted in down-regulation of N-cadherin and vimentin expression, and up-regulation of E-cadherin (Figure 3C). Immunofluorescence assays also confirmed that down-regulation of NEAT1 resulted in an increase in the epithelial marker and a decrease in the two mesenchymal markers in 786-O and ACHN cells (Figure 3D). Thus, down-regulation of NEAT1 inhibits RCC cell migration and invasion by suppressing EMT.

### Down-regulation of NEAT1 enhances RCC cell chemosensitivity

**Figure 4:**
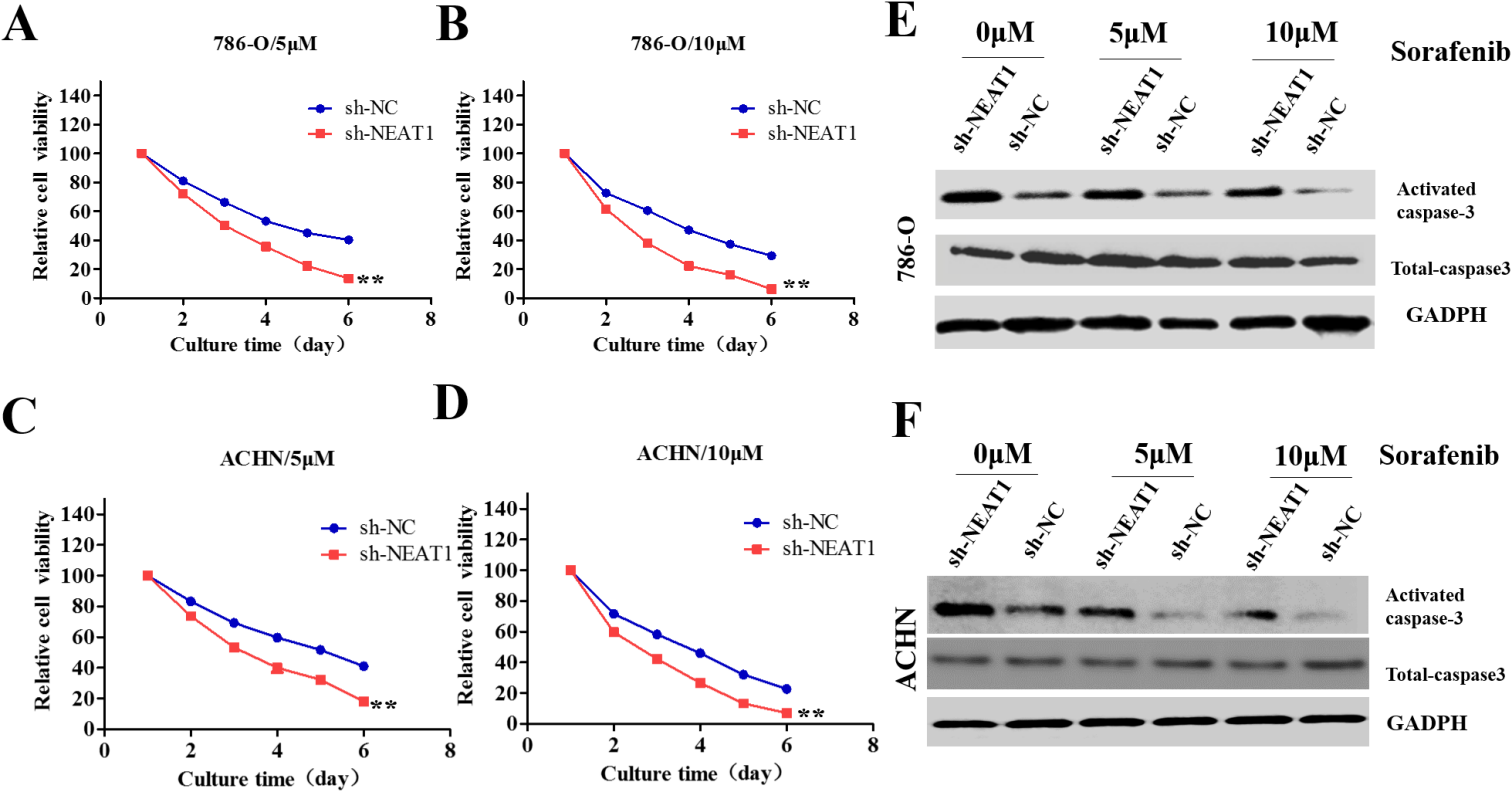
Role of NEAT1 in the sensitivity of RCC cells to sorafenib **(A-D)** CCK8 assays of NEAT1-knockdown or control cells incubated with the indicated concentrations of sorafinib. The error bars represent the mean ± SEM of at least three independent experiments. *p < 0.01 compared to controls. **(E** and **F)** Western blot of apoptosis-associated proteins (activated and total caspase-3). GAPDH was used as the loading control.

Sorafenib has been approved as a first- or second-line treatment for metastatic RCC [18]. We investigated whether knockdown of NEAT1 could enhance the sensitivity of RCC cells to sorafenib. Interestingly, sh-NEAT1 cells were more sensitive to sorafenib than control cells (Figure 4A-4D). Additionally, NEAT1 knockdown increased the expression of activated caspase-3 (a marker of apoptosis) in 786-O and ACHN cells, although the total proteins levels remained unchanged (Figure 4E and 4F).

### NEAT1 negatively regulates miR-34a to promote RCC progression

**Figure 5:**
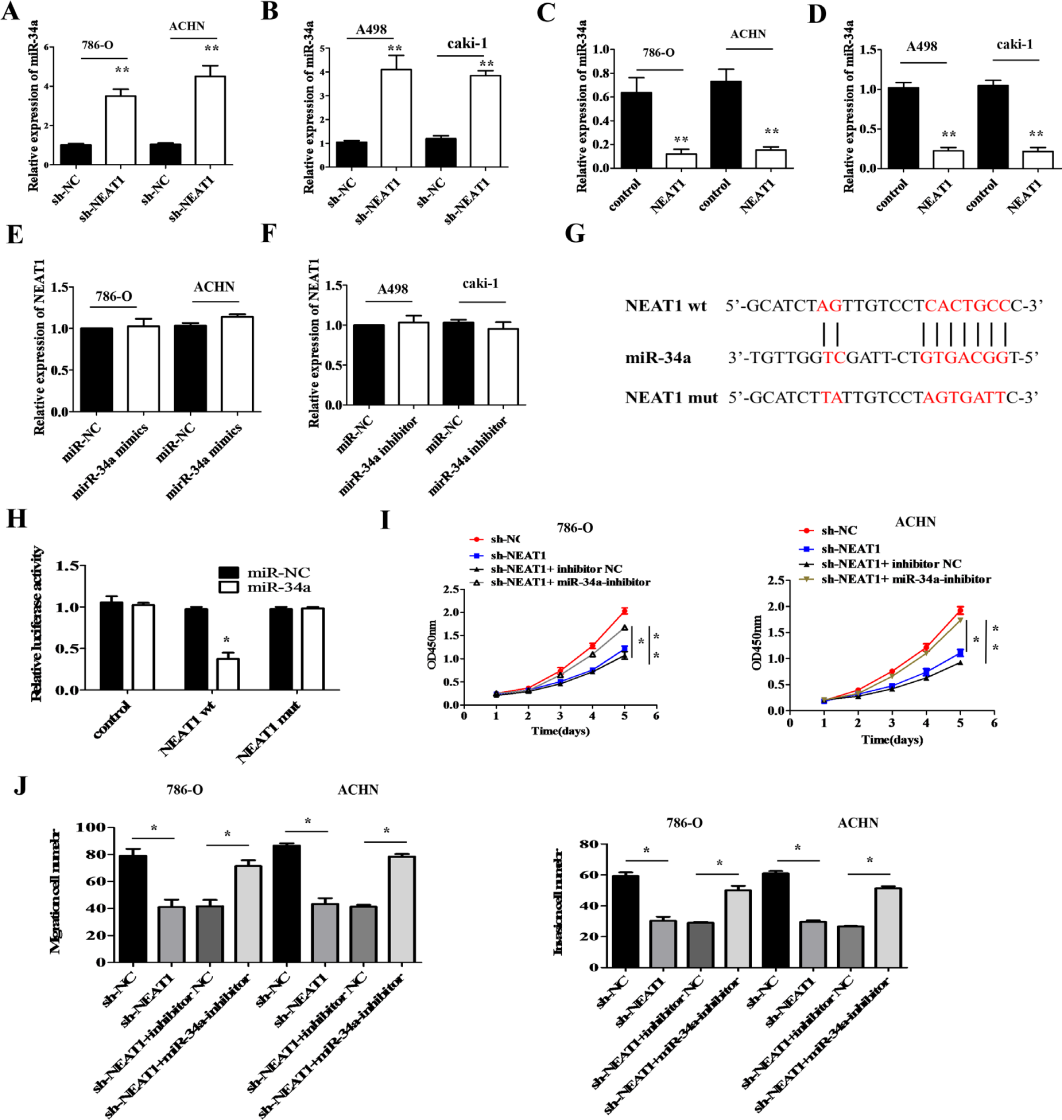
MiR-34a is a target of NEAT1 **(A** and **B)** The effects of NEAT1 knockdown on miR-34a levels was measured in RCC cells by qRT-PCR. *p < 0.01 compared to controls. **(C** and **D)** Measurement of miR-34a levels by qRT-PCR following over-expression of NEAT1 in RCC cells.*p < 0.01 compared to controls. **(E** and **F)** NEAT1 levels measured by qRT-PCR after over-expression or knockdown of miR-34a. **(G)** Bioinformatics analysis predicts miR-34a binding sites at two distinct positions in NEAT1. **(H)** Luciferase activity in RCC cells co-transfected with miR-34a mimics or a negative control and luciferase reporters containing no insert (control), NEAT1 wt, or NEAT1 mut. The error bars represent the mean ± SEM of at least three independent experiments. *p < 0.05 and *p < 0.01, respectively, compared to controls. **(I)** Analysis of the cell proliferation rate after co-transfection of 786-O and ACHN cells with NEAT1 shRNA and miR-34a inhibitors, using CCK-8 assays. *p < 0.05 and *p < 0.01, respectively, compared to controls. **(J)** MiR-34a inhibition abolishes the effects of the NEAT1 knockdown on the migration and invasion of 786-O and A498 cells. *p < 0.05 and *p < 0.01, respectively, compared to controls.

Many lncRNAs are known to function as competitive endogenous RNAs (ceRNAs) for specific miRNAs [19]. We used the starBase v2.0 software to identify miRNAs with complementary sequences to the NEAT1 transcript. Previous studies have indicated that miR-34a acts as a tumor suppressor in various cancers [20]. We therefore investigated whether miR-34a expression in RCC cells was altered in response to either NEAT1 knockdown or over-expression. MiR-34a expression was up-regulated in NEAT1 knockdown RCC cells compared to negative controls (Figure 5A and 5B; *p* < 0.01). In contrast, NEAT1 over-expression induced down-regulation of miR-34a expression in RCC cells compared to negative controls (Figure 5C and 5D; *p* < 0.01). No differences in NEAT1 levels were observed in response to ectopic expression or knockdown of miR-34a (Figure 5E and 5F).

Dual-luciferase reporter assays were performed to validate the association between NEAT1 and miR-34a. We constructed NEAT1 wild-type (wt) and mutant (mut) reporter vectors for these experiments (Figure 5G). Interestingly, miR-34a decreased the luciferase activity of the wt NEAT1 reporter vector but not that of the mut NEAT1 reporter (Figure 5H; *p* < 0.05). Rescue experiments were performed by co-transfecting NEAT1 shRNA and the miR-34a inhibitor into 786-O and ACHN cells. Inhibition of miR-34a abolished NEAT1 knockdown-induced suppression of cell proliferation in 786-O and ACHN cells compared to controls (Figure 5I; *p* < 0.05 and *p* < 0.01, respectively). In addition, NEAT1 knockdown-induced suppression of cell migration and invasion was effectively reversed by inhibition of miR-34a compared to controls (Figure 5J; *p* < 0.05). These data suggest that miR-34a is a direct target of NEAT1 in RCC cells, and that NEAT1 over-expression promotes RCC progression through inhibition of miR-34a.

### NEAT1 regulates the miR-34a/c-Met axis in RCC

**Figure 6:**
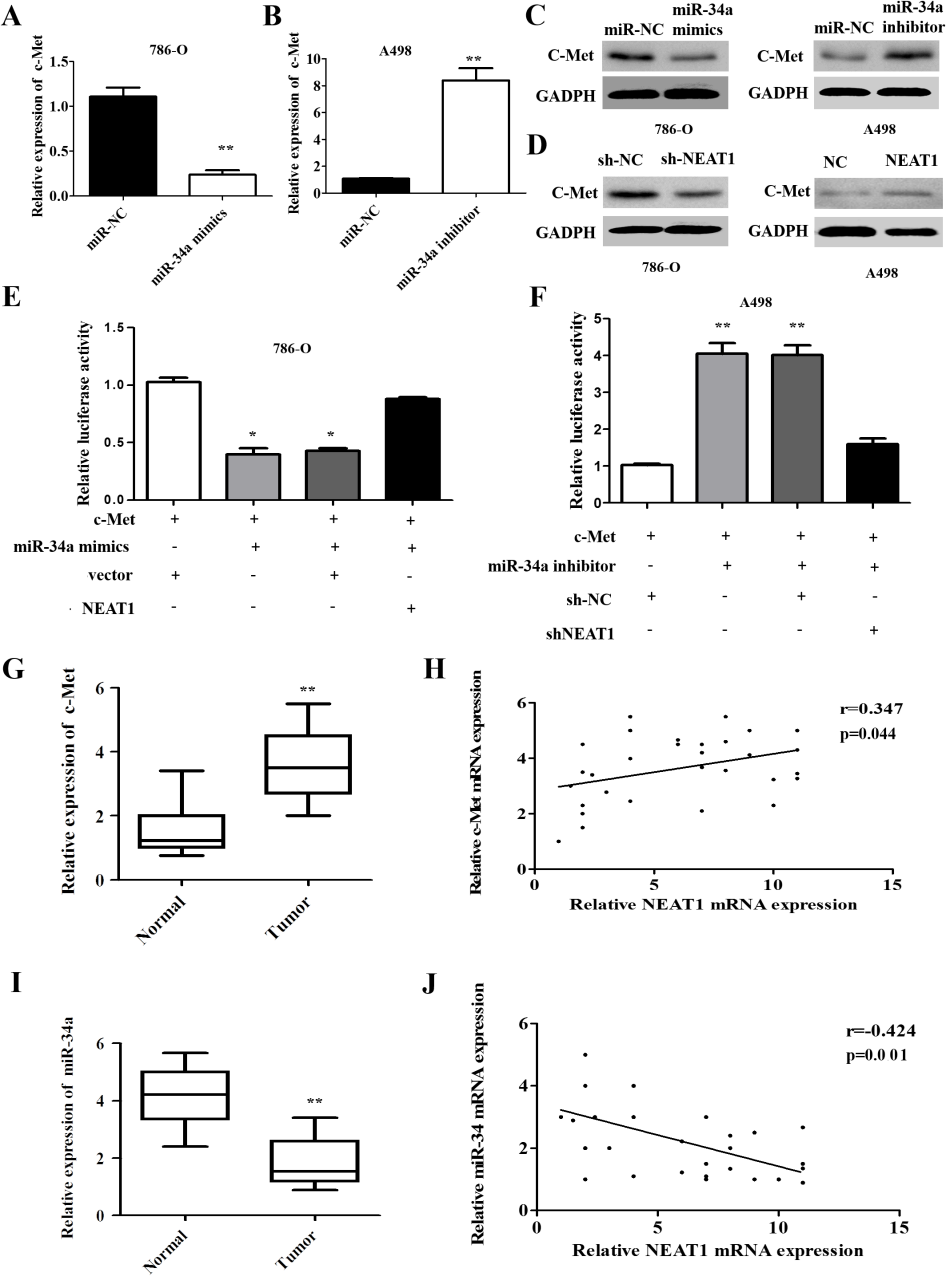
NEAT1 regulates the miR-34a/c-Met axis in RCC **(A)** NEAT1 knockdown results in down-regulation of c-Met mRNA in 786-O cells. **(B)** NEAT1 over-expression results in up-regulation of c-Met mRNA in A498 cells. **(C)** Western blot analysis of c-Met levels after miR-34a over-expression or knockdown. **(D)** Effect of aberrant NEAT1 expression on cMET protein levels as measured by Western blotting. **(E** and **F)** In the presence of p-NEAT1, c-Met expression was restored compared to controls **(E)** and reduced c-Met expression after NEAT1 silencing **(F)**. The error bars represent the mean ± SEM of at least three independent experiments. *p < 0.05 and *p < 0.01, respectively, compared to controls. **(G** and **H)** The expression of c-Met in 30 paired samples of primary RCC and normal tissue **(G)**, NEAT1 and c-Met expression are positively correlated in 30 RCC tissue samples (r = 0.347, *p* = 0.044; Figure 6H). **(I** and **J)** Analysis of miR-34a expression in 30 paired RCC and normal tissue samples by qRT-PCR **(I)**, NEAT1 and miR-34a expression are negatively correlated in 30 RCC tissue samples (r = −0.424, *p* = 0.001; Figure 6J).

Previous studies have demonstrated that miR-34a contributes to the progression of cancer by directly targeting c-Met [21]. We hypothesized that NEAT1 and c-Met interact with miR-34a by functioning as ceRNAs in RCC. Therefore, we examined c-Met expression in response to different levels of miR-34a. We found that dysregulation of miR-34a expression regulated c-Met at the mRNA and protein level in RCC cells (Figure 6A-6C; *p* < 0.01). We next investigated whether NEAT1 regulated c-Met expression in RCC cells. Indeed, NEAT1 knockdown reduced the levels of endogenous c-Met, while NEAT1 over-expression increased c-Met expression (Figure 6D). The c-Met vector was then co-transfected with miR-34a mimics and either p-NEAT1 or shNEAT1 into 786-O or A498 cells. These results indicated that the repression of c-Met expression was rescued in the presence of p-NEAT1 (Figure 6E). In contrast, the repression of c-Met was abolished in A498 cells after transfection with shNEAT1 (Figure 6F; *p* < 0.01).

We evaluated the expression of c-Met in 30 paired primary RCC and corresponding normal tissue samples using qRT-PCR. The relative levels of c-Met in RCC tissue were significantly higher than in corresponding normal tissue (Figure 6G; *p* < 0.01). Importantly, c-Met and NEAT1 levels were positively correlated (r = 0.347, *p* = 0.044; Figure 6F). We also examined the expression of miR-34a in 30 paired RCC and corresponding normal tissue samples using qRT-PCR, and found that miR-34a levels in RCC tissue were reduced in RCC compared to corresponding normal tissue (Figure 6I; *p* < 0.01). Additionally, miR-34a expression was negatively correlated with NEAT1 expression in RCC tissue (r = −0.424, *p* = 0.001; Figure 6J). These data suggest that NEAT1 regulates c-Met by functioning as a molecular sponge for miR-34a in RCC.

## DISCUSSION

NEAT1 is up-regulated in various cancers [22]. However, the functions of NEAT1 in RCC are not well understood. Here, we found that NEAT1 expression was up-regulated in RCC compared to adjacent normal tissue. Additionally, high NEAT1 expression was correlated with advanced stage, metastasis, and shorter overall survival after nephrectomy in RCC patients. These results suggest that NEAT1 may be a novel indicator of RCC patient prognosis.

High proliferative activity is a hallmark of cancer cells [23, 24]. NEAT1 has been previously associated with the growth of several cancers [12, 25]. For example, Chen et al. demonstrated that NEAT1 over-expression promoted the proliferation of esophageal squamous cell carcinoma cells [12]. We found that NEAT1 knockdown suppressed RCC cell growth by inhibiting cell cycle progression. These findings together with the high expression of NEAT1 in RCC tissue suggest that NEAT1 may function as an oncogene that promotes RCC progression.

Metastasis is the primary cause of mortality in RCC patients [26]. Previous studies have shown that NEAT1 silencing suppresses gastric cancer cell migration and invasion *in vitro* [27]. Consistent with these data, our results indicated that down-regulation of NEAT1 inhibited the migratory ability of RCC cells. EMT contributes to RCC metastasis [28]. We also demonstrated that NEAT1 knockdown resulted in down-regulation of vimentin and N-cadherin expression, and up-regulation of E-cadherin expression. These data suggest that NEAT1 is a potential therapeutic target in RCC.

Surgery in combination with chemotherapy is the standard of care for RCC [29]. Recently, sorafenib has emerged as a popular chemotherapeutic for RCC [18, 30]. However, sorafenib resistance is a significant clinical challenge. A more detailed understanding of the molecular mechanisms responsible for sorafenib resistance is required to facilitate the rational design of combination therapies that can block compensatory signaling pathways. We demonstrated that NEAT1 knockdown enhanced the sensitivity of RCC cells to sorafenib *in vitro*. Additionally, we showed that NEAT1 knockdown enhanced sorafenib-induced apoptosis in RCC cells. These results suggest that NEAT1-targeted therapies could be effective for the treatment of sorafenib-resistant RCC.

Recent evidence indicates lncRNAs may function as sponges for miRNAs and abolish the endogenous inhibitory effect of these miRNAs on their targets [31]. Indeed, NEAT1 has been implicated in the ceRNA network [32, 33]. Lu et al. demonstrated that NEAT1 promotes EMT and radioresistance in nasopharyngeal carcinoma by competitively sponging miR-204 [32]. We found that the sequence of NEAT1 aligned with the sequence of miR-34a and verified that NEAT1 was a target of miR-34a. Previous studies have reported that c-Met is a target of miR-34a [34]. Our results demonstrate that dysregulation of NEAT1 expression regulates c-Met at the mRNA and protein level in RCC cells. In addition, our data indicate that NEAT1 positively regulates the miR-34a target gene c-Met in RCC tissue. Thus, miR-34a is negatively regulated by NEAT1 in RCC cells, and NEAT1 serves as a ceRNA to up-regulate c-Met by sequestering miR-34a.

Although NEAT1-c-Met signaling is known to play a role in several cancers [35, 36], our findings are the first indication that NEAT1 contributes the progression of RCC through the miR-34a/c-Met axis. MiR-34a acts as a tumor suppressor in various cancers including RCC [37]. We demonstrated that NEAT1 functions as a ceRNA for miR-34a, which could be a novel diagnostic and therapeutic target for RCC.

## MATERIALS AND METHODS

### Clinical specimens

Primary RCC and normal renal tissue specimens were obtained from 102 patients at the Department of General Surgery, The Second Affiliated Hospital of Nanchang University, between May 2010 and January 2015. All specimens were obtained during radical or partial nephrectomy, immediately frozen in liquid nitrogen, and stored at −80°C until further analysis. The classification of tumor and normal tissues was confirmed by the pathologists. This study was performed with the approval of the Ethics and Research Committees of The Second Affiliated Hospital of Nanchang University. Written informed consent was obtained from all of the study participants.

### Cell culture

Human RCC cell lines (ACHN, 786-O, A498, and Caki-1) and normal human epithelial kidney cells (HK-2) were purchased from the American Type Culture Collection (ATCC, USA). All cells were cultured in F-12K or DMEM (Gibco, NY, USA) supplemented with 10% fetal bovine serum in a humidified atmosphere containing 5% CO_2_ at 37°C.

### Quantitative RT-PCR

Total RNA was isolated from tissues and cultured cells using the Trizol reagent (Invitrogen, USA) according to the manufacturer's instructions. RNA was reverse transcribed using the PrimeScript RT Reagent Kit (Invitrogen, USA) and quantitative real-time PCR (qRT-PCR) performed using the SYBR Premix Ex Taq (TaKaRa, China) according to the manufacturer's instructions. The primers were designed as follows: NEAT1, forward: 5′-TGGCTAGCTCAGGGCTTCAG-3′; reverse: 5′-TCTCCTTGCCAAGCT TCCTT-3′ and GAPDH, forward: 5′- CTGGGCTACACTGAGCACC-3′; reverse: 5′- AAGTGGTCGTTGAGGGCAATG-3′. The miR-34a primer (5′-TCTGTCTCTCTTG GCAGTGTCTT-3′) was purchased from RiboBio (China). Human U6 RNA was used as an internal miRNA control. The U6 primer was designed as follows: 5′-CTCGCTTCGGCAGCACA-3′. All reactions were performed in triplicate.

### Construction of NEAT1 knockdown or over-expressing stable cell lines

The lentivirus-encoding short hairpin RNA (Lv-shRNA) targeting NEAT1 (NR_131012) was constructed by GenePharma (China). The NEAT1 shRNA sequence was as follows: 5′-GCCATCAGCTTTGAATAAATT-3′. Synthetic sequence-scrambled shRNA (GenePharma) was used as a negative control. The human NEAT1 gene (NR_131012) was ligated into the pGCMV/MCS/RFP/Neo vector (GenePharma). Empty vector was used as a negative control. For the generation of stable cell lines, p-NEAT1 was transfected into RCC cells and selected with puromycin. The efficiency of knockdown or over-expression was assessed at the mRNA level using qRT-PCR.

### EdU assays

Cell proliferation assays were performed by incubating the cells with 5-ethynyl-20-deoxyuridine (EdU) (Invitrogen, USA) for 5 hours followed by incubation with 300 μL of 1 × Apollo® reaction cocktail for 30 minutes. The cells were stained with Hoechst 33342 for 30 minutes and nuclei visualized under a fluorescence microscope.

### Cell proliferation, migration, and invasion assays

Cell proliferation, migration, and invasion assays were performed as described previously [15]. All experiments were repeated three times.

### Cell cycle assays

Cells were seeded into six-well plates at a density of 1 × 10^5^ cells/well. After 48 hours, the cells were fixed in 70% ethanol at 4°C overnight. Cell cycle analysis was performed using the Cycletest™ Plus DNA Reagent Kit (BD Biosciences, USA) according to the manufacturer's protocol.

### Western blot analysis

Western blot analysis was performed as described previously [16]. The primary antibodies were the following: anti-E-cadherin (1:1000, Cell Signaling Technology, USA), anti-N-cadherin (1:1000, Cell Signaling Technology), anti-Vimentin (1:1000, Abcam, USA), anti-activated caspase-3 (1:1000, Cell Signaling Technology), anti-total caspase-3 (1:1000, Cell Signaling Technology), anti-c-Met (1:1000, Abcam), and anti-GAPDH (1:1000, Santa Cruz Biotechnology, USA).

### Immunofluorescence analysis

A total of 5 × 10^3^ cells were cultured on coverslips for 24 hours, fixed with paraformaldehyde for 30 minutes, and then permeabilized with 0.3% Triton X-100/phosphate-buffered saline for 5 minutes at room temperature. The cells were then blocked with 5% bovine serum albumin for 1 hour at room temperature, stained with anti-E-cadherin (1:200, Cell Signaling Technology) and anti-N-cadherin (1:200, Cell Signaling Technology) antibodies at 4°C overnight, incubated withafluorescent dye-conjugated secondary antibody (1:200, Invitrogen, USA) for 1 hour, and then stained with DAPI. Fluorescence images were collected using a confocal microscope.

### Vector construction and luciferase reporter assays

The miR-34a inhibitor and miR-34a mimic were purchased from RiboBio (China). The pcDNA-c-Met vector was constructed by inserting c-Met cDNA in the pcDNA vector (GenePharma). NEAT1 3′ UTR-WT and NEAT1 3′ UTR-MUT were cloned into pmirGLO luciferase plasmids (GenePharma), which were transfected into RCC cells using Lipofectamine 2000 (Invitrogen). Luciferase activity was measured after 48 hours using the Dual-Luciferase Reporter Assay System (Promega, China) according to the manufacturer's instructions. All experiments were repeated three times.

### Statistical analysis

All results are shown as the mean ± standard error of the mean (SEM). The data from at least three independent experiments were analyzed using the GraphPad Prism 5 software (GraphPad, USA). Two-tailed paired Student's t-tests and one-way ANOVA were used to compare different groups. A *p* < 0.05 was considered statistically significant.

## Abbreviations

lncRNAs: long non-coding RNAs
NEAT1: nuclear enriched abundant transcript 1
RCC: renal cell carcinoma
EMT: epithelial-to-mesenchymal transition
ceRNAs: competitive endogenous RNAs
mut: mutant
RT-PCR: reverse transcription polymerase chain reaction
qRT-PCR: quantitative RT-PCR.
